# Incidence and Predictors of Cardiac Arrhythmias in Patients With COVID-19

**DOI:** 10.3389/fcvm.2022.908177

**Published:** 2022-06-22

**Authors:** Sahar Mouram, Luigi Pannone, Anaïs Gauthey, Antonio Sorgente, Pasquale Vergara, Antonio Bisignani, Cinzia Monaco, Joerelle Mojica, Maysam Al Housari, Vincenzo Miraglia, Alvise Del Monte, Gaetano Paparella, Robbert Ramak, Ingrid Overeinder, Gezim Bala, Alexandre Almorad, Erwin Ströker, Juan Sieira, Pedro Brugada, Mark La Meir, Gian Battista Chierchia, Carlo de Asmundis

**Affiliations:** ^1^Heart Rhythm Management Centre, Postgraduate Program in Cardiac Electrophysiology and Pacing, Universitair Ziekenhuis Brussel - Vrije Universiteit Brussel, European Reference Networks Guard-Heart, Brussels, Belgium; ^2^Department of Cardiac Surgery, Universitair Ziekenhuis Brussel - Vrije Universiteit Brussel, Brussels, Belgium

**Keywords:** COVID-19, SARS-CoV-2, CT severity score, cardiac arrhythmias, pulmonary damage

## Abstract

**Background:**

Coronavirus disease 2019 (COVID-19) is a systemic disease caused by severe acute respiratory syndrome coronavirus 2. Arrhythmias are frequently associated with COVID-19 and could be the result of inflammation or hypoxia. This study aimed to define the incidence of arrhythmias in patients with COVID-19 and to correlate arrhythmias with pulmonary damage assessed by computed tomography (CT).

**Methods:**

All consecutive patients with a COVID-19 diagnosis hospitalized at Universitair Ziekenhuis Brussel, Belgium, between March 2020 and May 2020, were screened. All included patients underwent a thorax CT scan and a CT severity score, a semiquantitative scoring system of pulmonary damage, was calculated. The primary endpoint was the arrhythmia occurrence during follow-up.

**Results:**

In this study, 100 patients were prospectively included. At a mean follow-up of 19.6 months, 25 patients with COVID-19 (25%) experienced 26 arrhythmic episodes, including atrial fibrillation in 17 patients, inappropriate sinus tachycardia in 7 patients, atrial flutter in 1 patient, and third-degree atrioventricular block in 1 patient. No ventricular arrhythmias were documented. Patients with COVID-19 with arrhythmias showed more often need for oxygen, higher oxygen maximum flow, longer QTc at admission, and worse damage at CT severity score. In univariate logistic regression analysis, significant predictors of the primary endpoint were: the need for oxygen therapy (odds ratio [*OR*] 4.59, 95% *CI* 1.44–14.67, *p* = 0.01) and CT severity score of pulmonary damage (*OR* per 1 point increase 1.25, 95% *CI* 1.11–1.4, *p* < 0.001).

**Conclusions:**

In a consecutive cohort of patients with COVID-19 the incidence of cardiac arrhythmias was 25%. The need for oxygen therapy and CT severity score were predictors of arrhythmia occurrence during follow-up.

## Introduction

Coronavirus disease 2019 (COVID-19) is a systemic disease caused by severe acute respiratory syndrome coronavirus 2 (SARS-CoV-2) that has been affecting millions of people around the world since December 2019 (1, 2). SARS-CoV-2 can cause damage to multiple organs (3). Detailed mapping of the viral RNA in tissues of 11 deceased patients with COVID-19 has shown cardiovascular dissemination of the virus in all patients (4).

Arrhythmias are one of the most common cardiovascular complications of SARS-CoV-2 dissemination (5, 6). This is not surprising, as acute infections are known to be a potential cardiovascular disease trigger (7). Arrhythmias are related to the severity of COVID-19 disease and could be the result of direct or indirect damage to myocardial tissue caused by multiple mechanisms (8). Pulmonary damage and its progression determine the prognosis during COVID-19 infection (9) and inflammation in pulmonary infections has been described as a trigger factor for arrhythmias (10).

Since the beginning of the COVID-19 pandemic, the incidence of cardiac arrhythmias has been described in different reports.

Bhatla et al. (11) described a cohort of 700 patients with 9 cardiac arrests, 25 incident atrial fibrillation (AF) events, 9 clinically significant bradyarrhythmias, and 10 non-sustained ventricular tachycardias. In a worldwide survey of COVID-19 including 4,526 hospitalized patients with COVID-19, 18% of patients developed an arrhythmia (12). Wang et al. (13) described a case series of 138 patients with 16.7% of patients developing arrhythmias. Finally in a survey by heart rhythm society, AF was the most common tachyarrhythmia (21%), while sinus bradycardia (8%) and atrio ventricular block (8%) were the most common bradyarrhythmias (14). However, the correlation between pulmonary damage and arrhythmias during COVID-19 infection is not well established.

The aim of this study is to define the global incidence of arrhythmias in patients with COVID-19 at follow-up; furthermore, a correlation between arrhythmia occurrence and the pulmonary damage induced by COVID-19 infection is sought.

## Methods

### Study Population

Between March 2020 and May 2020, all consecutive patients with COVID-19 diagnosis, hospitalized at Universitair Ziekenhuis Brussel, Belgium, were prospectively screened for enrollment in the study and followed-up.

Inclusion criteria were: (1) positive nasopharyngeal swab for SARS-CoV-2 test with real-time PCR (RT-PCR) assay and (2) lung computed tomography (CT) scan performed and CT scan severity score calculated and available (15, 16).

Exclusion criteria were: (1) previous history of cardiac arrhythmias; (2) previous history of myocardial infarction or of any cardiomyopathy; and (3) COVID-19 associated myocarditis diagnosis following current guidelines (17).

Clinical and laboratory data were collected on a shared electronic medical record. Arrhythmia occurrence was defined as at least one of the following: AF, diagnosed following current guidelines (18), inappropriate sinus tachycardia (IST) (19), atrial flutter, third-degree atrioventricular block (AVB), premature ventricular complexes, monomorphic or polymorphic ventricular tachycardia, and ventricular fibrillation. Deaths were collected on a daily basis and causes of death were recorded. Clinical data were extracted from the electronic medical records and merged with the ECG data. Data were carefully reviewed and confirmed by 2 independent researchers (S.M. and L.P.), both blinded to the occurrence of cardiac arrhythmia and guarantee the accuracy of the data extraction.

A standard 12-lead ECG (20) was performed at admission and daily for all patients during hospitalization. Patients hospitalized in intensive care unit (ICU) underwent continuous ECG monitoring. Data were analyzed by 2 independent physicians (S.M. and L.P.) who were blinded to follow-up, and if there was a discrepancy of interpretation, a third reviewer arbitrated (C.d.A.) (also blinded to follow-up). The following parameters were collected and analyzed on the first ECG at admission: PQ interval (ms), QRS interval (ms), and QTc interval (ms) with the Bazett formula in lead DII (21).

Furthermore, data on medical history, cardiac implantable electronic devices, ICU admission, oxygen requirement, echocardiography, and drug treatment were collected and analyzed.

The study complied with the Declaration of Helsinki, as revised in 2013.

### CT Scan and CT Scan Severity Score

At admission, all patients underwent a CT scan with a CT 256-Slice Scanner (GE Healthcare system). All chest CT scans were assessed at lung and mediastinal window using 2D coronal and sagittal planes. Two experienced radiologists, both blinded to clinical follow-up, performed the CT image analysis. A CT severity score, a semiquantitative scoring system of pulmonary damage, was calculated. A CT severity score was determined based on lobar damage degree, as previously described (15, 16). Each of the five lobes was scored on a scale of 0–5, with 0 indicating no damage, 1 point indicating <5% damage, 2 points for 5–25% damage, 3 points for 26–49% damage, 4 points for 50–75% damage, and 5 points for more than 75% damage. The total CT severity score was the sum of lobar scores, ranging from 0 to 25.

### Follow-Up

The date of inclusion in the study was defined as the date of the first positive nasopharyngeal swab for SARS-CoV-2 during index hospitalization. Patients were followed up in outpatient clinic every 6 months with a standard 12-lead ECG and a 24-h ECG Holter. In the case of symptoms, a 12-lead ECG and a 24-h ECG Holter were performed. The primary endpoint was any arrhythmia occurrence, defined as at least one of the following: AF diagnosed following current guidelines (18), IST (19), atrial flutter, third-degree atrioventricular block, premature ventricular complexes, monomorphic or polymorphic ventricular tachycardia, and ventricular fibrillation. Deaths and cause of deaths were collected and analyzed.

### Statistical Analysis

All variables were tested for normality with the Shapiro–Wilk test. Normally distributed variables were described as mean ± standard deviation (SD) and the groups were compared through ANOVA, paired or unpaired *t*-test as appropriate, while the non-normally distributed variables were described as median (interquartile range [IQR]) and compared by the Mann–Whitney test or Wilcoxon signed-rank test as appropriate. The categorical variables were described as frequencies (percentages) and compared by the chi-squared test or Fisher's exact test as appropriate. Cohen's kappa statistic was used to assess interobserver agreement in ECG analysis.

A logistic regression analysis was performed to predict arrhythmia occurrence during follow-up. The variables included in the univariate logistic regression model were as follows: age, gender, QTc interval at admission, C-reactive protein (CRP) at admission, need for oxygen therapy, CT severity score of pulmonary damage. For each significant predictor, the odds ratio (*OR*) and relative 95% confidence interval (*CI*) were calculated. The best cutoff for each significant continuous variable was obtained using the Youden's method (22). Sensitivity and specificity were calculated at the best cutoff. Discrimination was measured by area under the receiver operating characteristic (ROC) curve measure (AUC).

Continuous variables, if significant predictors at logistic regression, were dichotomized based on the cutoff derived by Youden's method.

Multivariate regression analysis was not performed because of the low number of events at follow-up; only univariate logistic regression analysis was performed to avoid overfitting problems (23).

Kaplan-Meier's curves were drawn to describe the patients' freedom from arrhythmias during the follow-up period and a log-rank test was used.

A value of *p* < 0.05 was considered statistically significant.

Survival analysis was performed with the *survival* (24) and *survminer* (25) packages on R software.

The analysis was performed using R software version 3.6.2 (R Foundation for Statistical Computing, Vienna, Austria).

## Results

### Study Population Characteristics

In this study, 100 consecutive patients were prospectively included and analyzed.

Complete patient characteristics are summarized in Table 1.

**Table 1:** Clinical characteristics of coronavirus disease 2019 (COVID-19) patients with and without a arrhythmias.

	**No arrhythmias (*N =* 75)**	**Any arrhythmia (*N =* 25)**	**Total (*N* = 100)**	***p*** **-value**
Age (years)	67.5 ± 18.2	73.7 ± 14.2	69.1 ± 17.4	0.12
Gender (male)	38 (50.7%)	12 (48.0%)	50 (50.0%)	1.00
Diabetes (*n*, %)	11 (14.7%)	6 (24.0%)	17 (17.0%)	0.36
Dyslipidemia (*n*, %)	11 (14.7%)	5 (20.0%)	16 (16.0%)	0.54
OSAS (*n*, %)	2 (2.7%)	1 (4.0%)	3 (3.0%)	1.00
AHT (*n*, %)	15 (20.0%)	7 (28.0%)	22 (22.0%)	0.41
Myocarditis (*n*, %)	0 (0.0%)	0 (0.0%)	0 (0.0%)	NA
Need of oxygen (*n*, %)	40 (53.3%)	21 (84.0%)	61 (61.0%)	0.009
Oxygen max (l/min)	3.2 ± 5.0	5.7 ± 6.3	3.8 ± 5.5	0.047
Pulmonary embolism (*n*, %)	0 (0.0%)	1 (4.0%)	1 (1.0%)	0.25
CT severity score of pulmonary damage	7.2 ± 5.1	13.2 ± 5.4	8.6 ± 5.8	<0.001
Type of arrhythmia				
AV block (*n*, %)	0 (0.0%)	1 (4.0%)	1 (1.0%)	
Inappropriate sinus tachycardia (*n*, %)	0 (0.0%)	7 (28.0%)	7 (7.0%)	
Duration of IST (months)	NA	4.7 ± 2.4	4.7 ± 2.4	
Heart rate of IST (bpm)	NA	107.7 ± 10.8	107.7 ± 10.8	
Sinus node dysfunction (*n*, %)	0 (0.0%)	0 (0.0%)	0 (0.0%)	
AF (*n*, %)	0 (0.0%)	17 (68.0%)	17 (17%)	
AF max rate (bpm)	NA	128.1 ± 37.8	128.1 ± 37.8	
Flutter (*n*, %)	0 (0.0%)	1 (4.0%)	1 (1.0%)	
ECG PR (ms)	158.5 ± 38.6	154.9 ± 29.9	158.0 ± 37.2	0.77
ECG QRS (ms)	90.0 ± 19.0	93.2 ± 16.1	90.9 ± 18.2	0.46
ECG QTc admission (ms)	444.6 ± 42.6	462.5 ± 46.5	449.5 ± 44.2	0.042
ECG QTc at CRP peak (ms)	474.6 ± 51.8	483.0 ± 49.0	470.5 ± 51.3	0.09
LVEF (%)	58.2 ± 7.0	51.7 ± 14.9	54.9 ± 11.8	0.17
CRP level admission (mg/L)	62.3 ± 55.4	84.5 ± 44.2	76.4 ± 48.3	0.037
CRP level peak (mg/L)	123.0 ± 96.8	120.2 ± 78.2	122.2 ± 91.5	0.90
Treatment				
Hydroxychloroquine (*n*, %)	28 (37.3%)	12 (48.0%)	40 (40.0%)	0.36
Antibiotics (*n*, %)	23 (31.1%)	10 (40.0%)	33 (33.3%)	0.47

*AF, atrial fibrillation; AHT, arterial hypertension; AV block, third degree atrioventricular block; ECG, electrocardiogram; EPS, electrophysiological study; LVEF, left ventricular ejection fraction (echocardiography); OSAS, obstructive sleep apnea syndrome; CRP, C-reactive protein; IST, inappropriate sinus tachycardia*.

The mean follow-up was 19.6 months ± 7.5. Twenty-five patients with COVID-19 (25%) experienced an arrhythmia. Compared with patients without any arrhythmia, patients with COVID-19 with arrhythmias showed more often need for oxygen therapy [21 patients (84.0%) vs. 40 patients (53.3%), *p* = 0.009], higher oxygen maximum flow [5.7 L/min ± 6.3 vs. 3.2 L/min ± 5.0, *p* = 0.047], higher CRP at admission [84.5 mg/L ± 44.2 vs. 62.3 mg/L ± 55.4, *p* = 0.037], longer QTc at admission [462.5 ms ± 46.5 vs. 444.6 ms ± 42.6, *p* = 0.042], and more extensive pulmonary involvement at CT severity score [13.2 ± 5.4 vs. 7.2 ± 5.1, *p* < 0.001]. There was no difference in drug treatment between the 2 groups (*p* = NS). Three patients (3%) had a cardiac implantable electronic device [3 patients (3%) had a pacemaker and no patients had an implanted loop recorder].

Good interobserver agreement was observed for ECG analysis (κ = 0.97).

### Follow-Up and Predictors of Arrhythmia Occurrence

At follow-up, there were 11 deaths (11%). Death occurred at a mean follow-up of 11.3 days ± 16.5 and the cause of death was adjudicated as respiratory insufficiency in all patients. There were no arrhythmic deaths. Eighteen patients (18%) were admitted to ICU.

**Figure 1:**
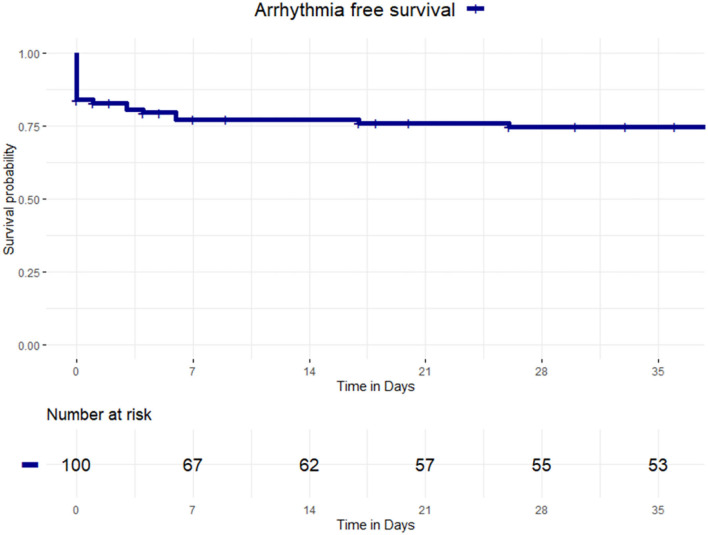
Kaplan-Meier curves of survival free from arrhythmia occurrence. Kaplan-Meier curve of survival free from any arrhythmia in patients with coronavirus disease 2019 (COVID-19).

At follow-up, 25 patients with COVID-19 (25%) experienced 26 arrhythmic episodes, occurring at a mean follow-up of 5.2 days ± 13.7 (Figure 1). The type of arrhythmia occurred was as follows: AF in 17 patients, IST in 7 patients, atrial flutter in 1 patient, and third-degree AVB in 1 patient. One patient experienced both IST and atrial flutter. The mean duration of IST after the first positive swab test for COVID-19 was 4.7 months ± 2.4. No ventricular arrhythmias occurred, including: premature ventricular complexes, monomorphic and polymorphic ventricular tachycardia, and ventricular fibrillation.

At univariate logistic regression analysis, significant predictors of primary endpoint were the following: need for oxygen therapy (*OR* 4.59, 95% *CI* 1.44–14.67, *p* = 0.01) and CT severity score of pulmonary damage (*OR* per 1 point increase 1.25, 95% *CI* 1.11–1.4, *p* < 0.001) (Table 2).

**Table 2:** Univariate logistic regression analysis for primary endpoint of any arrhythmia occurrence.

	**Univariate logistic regression analysis OR (CI 95%), *p*-value**
Age	1.02 (0.99–1.05), *p =* 0.13
Gender (male)	0.9 (0.36–2.22), *p =* 0.82
QTc admission	1.1 (0.99–1.2), *p =* 0.10
CRP level admission	1.1 (0.99–1.1), *p =* 0.21
Need for oxygen therapy	4.59 (1.44–14.67), *p =* 0.01
CT severity score	1.25 (1.11–1.4), *p* < 0.001

The best cutoff at CT severity score to predict arrhythmia occurrence was 10.5 (specificity 0.78, sensitivity 0.82, and AUC 0.75). Thirty-six patients (36%) had a CT score ≥10.5.

**Figure 2:**
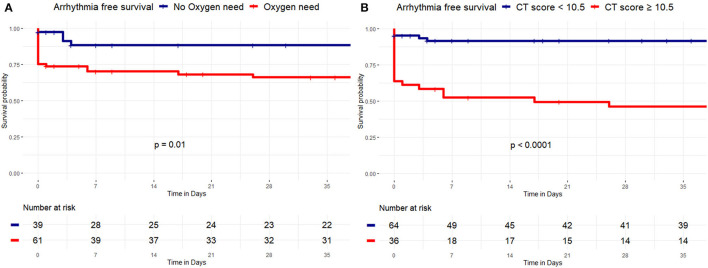
Kaplan-Meier curves of survival free from arrhythmia occurrence by oxygen need and a CT severity score. Kaplan-Meier curve of survival free from arrhythmias by oxygen therapy need and a CT severity score. **(A)** Patients with no oxygen therapy need (blue curve) had higher VA free survival during the follow-up, compared with patients with oxygen therapy need (red curve), (89.7 vs. 65.6%, Log-Rank *p* = 0.01). **(B)** Patients with CT severity score < 10.5 (blue curve) had higher VA free survival during the follow-up, compared with patients with CT severity score ≥ 10.5 (red curve), (90.6 vs. 47.2%, Log-Rank *p* < 0.001).

Figure 2 shows that patients with COVID-19 who did not require oxygen therapy had higher arrhythmia free survival during the follow-up compared with patients who needed oxygen (89.7 vs. 65.6%, Log-Rank *p* = 0.01).

Furthermore, patients with COVID-19 with a CT severity score < 10.5 showed higher arrhythmia free survival during the follow-up compared with patients with CT score ≥ 10.5 (90.6 vs. 47.2%, Log-Rank *p* < 0.001) (Figure 2).

## Discussion

The main findings of this study are the following: (1) in a consecutive cohort of patients with COVID-19, the incidence of cardiac arrhythmias, such as both brady- and tachyarrhythmias was 25%; (2) patients with arrhythmias showed a worse inflammatory status as demonstrated by higher CRP and more extensive pulmonary involvement; (3) the need of oxygen therapy and CT severity score were predictors of arrhythmia occurrence during a mean follow-up of 19 months.

### Arrhythmic Burden in COVID-19

Cardiac arrhythmias are a well-known and serious complication of COVID-19 infection. In the literature, there is conflicting data on the incidence, with a reported occurrence in 7–44% of all patients with COVID-19 (6). This is consistent with the heterogeneity of population enrolled. Indeed, in clinically stable patients, without need for ICU stay, arrhythmic episodes were detected in only 9% of cases (26). AF was the most common arrhythmia diagnosed (6%). In patients with COVID-19 hospitalized in ICU, the incidence rate of cardiac arrhythmias was found to be higher, up to 44% (13, 27). In the current study, the reported arrhythmic incidence rate of 25% is consistent with previous reports and reflects a population with an ICU admission needed in 18% of patients. Furthermore, this study confirmed AF as the most common incident arrhythmia in patients with COVID-19. Previous data were hampered by focusing mostly on one type of arrhythmias, such as tachy- or bradyarrhythmias (28) and by short-term follow-up (26). The current study provides a global snapshot of arrhythmic burden in patients with COVID-19, including third-degree AVB that was previously reported only in case reports (29). Furthermore, the follow-up hereby described (19.6 months), has allowed to provide an important information related to the mean duration of IST after a COVID 19 infection requiring hospitalization, which showed to be 4.7 months.

### Predictors of Cardiac Arrhythmias and the Role of Pulmonary Inflammation

Different mechanisms have been proposed as putative for arrhythmogenesis in patients with COVID-19 including inflammation and hypoxia secondary to lung injury (6).

Systemic inflammation has been demonstrated as proarrhythmic in acute infections: in particular, cytokines, such as interleukin-6 and interleukin-1, cause QTc prolongation secondary to increased expression of K+ channel gene KCNJ2 (30). In patients with COVID-19, QTc interval at admission were demonstrated to correlate with CRP levels, reflecting the inflammatory status (31). Consistent with these findings, in the current study, patients with arrhythmias had prolonged QTc at admission and higher CRP level at admission compared to patients without arrhythmias. CRP has not been demonstrated as a predictor at univariate logistic regression analysis, probably because of the low number of events.

Lung injury in COVID-19 is associated with hypoxia-induced damage in myocardial cells. Specifically, hypoxia-mediated anaerobic metabolism increases cytosolic calcium levels. This can trigger early and late depolarizations and temporal alterations in the action potential duration (32). Furthermore, hypoxia increases the extracellular potassium levels and can determine a reduced electrical coupling, mediated by connexin 43 in gap junctions (33). Building upon these premises, the need of oxygen therapy and CT severity score were predictors of arrhythmia occurrence in the current study. This may reflect a severe hypoxia-induced cellular damage triggering cardiac arrhythmias.

## Limitations

Limitations include referral bias due to the inclusion of study patients from a tertiary center. Multivariate regression analysis was not performed because of the low number of events at follow-up. Thus, the low sample size does not enable to adjust for confounding factors. All arrhythmic events occurred before discharge. Validation of the CT severity score, prospectively and in large COVID-19 cohorts, would add value to its clinical utility in cardiac arrhythmias prediction. Myocarditis may contribute to arrhythmogenesis in COVID-19; however, it was an exclusion criteria in the current study.

## Conclusions

In a consecutive cohort of patients with COVID-19, the incidence of cardiac arrhythmias was 25%. The need of oxygen therapy and CT severity score were predictors of arrhythmia occurrence at 19 months follow-up.

## Data Availability Statement

The raw data supporting the conclusions of this article will be made available by the authors, without undue reservation.

## Ethics Statement

The studies involving human participants were reviewed and approved by Commissie Medische Ethiek UZ Brussel. The patients/participants provided their written informed consent to participate in this study.

## Author Contributions

Conception and design of the work and drafting the work: SM and LP. Substantial contributions to the acquisition of data for the work: SM, LP, AG, AS, PV, AB, CM, JM, MA, VM, GP, RR, and IO. Substantial contributions to the analysis of data for the work: SM, LP, AG, AS, and PV. Substantial contributions to the interpretation of data for the work, final approval of the version to be published, and agreement to be accountable for all aspects of the work in ensuring that questions related to the accuracy or integrity of any part of the work are appropriately investigated and resolved: SM, LP, AG, AS, PV, AB, CM, JM, MA, VM, AD, GP, RR, IO, GB, AA, ES, JS, PB, ML, GC, and CA. Revising the draft of the work critically for important intellectual content: GB, AA, ES, JS, PB, ML, GC, and CA. All authors contributed to the article and approved the submitted version.

## Conflict of Interest

AB is a consultant for Biotronik. VM received an educational grant from the Foundation “Enrico and Enrica Sovena”. PB received compensation for teaching purposes from Biotronik. ML is consultant for Atricure. GC received compensation for teaching purposes and proctoring from Medtronic, Abbott, Biotronik, Boston Scientific, and Acutus Medical. CA receives research grants on behalf of the center from Biotronik, Medtronic, Abbott, LivaNova, Boston Scientific, AtriCure, Philips, and Acutus; compensation for teaching purposes and proctoring from Medtronic, Abbott, Biotronik, Livanova, Boston Scientific, Atricure, and Acutus Medical Daiichi Sankyo. The remaining authors declare that the research was conducted in the absence of any commercial or financial relationships that could be construed as a potential conflict of interest.

## Publisher's Note

All claims expressed in this article are solely those of the authors and do not necessarily represent those of their affiliated organizations, or those of the publisher, the editors and the reviewers. Any product that may be evaluated in this article, or claim that may be made by its manufacturer, is not guaranteed or endorsed by the publisher.
